# Time trends and heterogeneity in the disease burden of visual impairment due to cataract, 1990–2019: A global analysis

**DOI:** 10.3389/fpubh.2023.1140533

**Published:** 2023-04-03

**Authors:** Xiaotong Han, Minjie Zou, Zhenzhen Liu, Yi Sun, Charlotte Aimee Young, Danying Zheng, Guangming Jin

**Affiliations:** ^1^State Key Laboratory of Ophthalmology, Zhongshan Ophthalmic Center, Guangdong Provincial Key Laboratory of Ophthalmology and Visual Science, Guangdong Provincial Clinical Research Center for Ocular Diseases, Sun Yat-sen University, Guangzhou, China; ^2^Department of Ophthalmology, Third Affiliated Hospital of Sun Yat-sen University, Guangzhou, China; ^3^Albany Medical College, Albany, NY, United States

**Keywords:** cataract, disease burden, disability-adjusted life year, global eye health, prevalence

## Abstract

**Objectives:**

This study aimed to estimate the disease burden of cataract and evaluate the contributions of risk factors to cataract-associated disability-adjusted life years (DALYs).

**Materials and methods:**

Prevalence and DALYs of visual impairment due to cataract were extracted from the Global Burden of Disease (GBD) study 2019 to explore time trends and annual changes. Regional and country-level socioeconomic indexes were obtained from open databases. The time trend of prevalence and DALYs was demonstrated. Stepwise multiple linear regression was used to evaluate associations between the age-standardized rate of DALYs of cataract and potential predictors.

**Results:**

Global Prevalence rate of visual impairment due to cataract rose by 58.45% to 1,253.9 per 100,000 population (95% CI: 1,103.3 to 1,417.7 per 100,000 population) in 2019 and the DALYs rate rose by 32.18% from 65.3 per 100,000 population (95% CI: 46.4 to 88.2 per 100,000 population) in 1990 to 86.3 per 100,000 population (95% CI: 61.5 to 116.4 per 100,000 population) in 2019. Stepwise multiple linear regression model showed that higher refractive error prevalence (β = 0.036, 95% CI: 0.022, 0.050, *P* < 0.001), lower number of physicians per 10,000 population (β = −0.959, 95% CI: −1.685, −0.233, *P* = 0.010), and lower level of HDI (β = −134.93, 95% CI: −209.84, −60.02, *P* = 0.001) were associated with a higher disease burden of cataract.

**Conclusion:**

Substantial increases in the prevalence of visual impairment and DALYs of cataract were observed from 1990 to 2019. Successful global initiatives targeting improving cataract surgical rate and quality, especially in regions with lower socioeconomic status, is a prerequisite to combating this growing burden of cataract in the aging society.

## Introduction

A cataract is the leading cause of blindness globally and has been a primary focus of many national programs aimed at meeting Vision 2020 objectives (1). Despite being treatable with a straightforward and cost-effective surgery, one of the greatest challenges facing ophthalmology globally today remains the unacceptably high backlog of operable cataract blindness, especially in the developing world (2, 3). It is reported that in 2015, cataract contributed to 55% of blindness among adults aged 50 years and older (4). With the increase in the aging global population, more people will be at risk for this common cause of vision loss in the coming years (5, 6). According to the World Health Organization (WHO) report on Vision 2019, there are at least 1 billion people with preventable moderate or severe distance vision impairment or blindness, including 65.2 million caused by cataract (5).

Although many breakthroughs have been made since the inception of Vision 2020 and decreasing blindness prevalence has been achieved during the past decades, the number of blind people continues to increase rapidly (4, 7). The rates of cataract surgery are increasing globally and postoperative outcomes are improving, yet challenges to reducing the cataract burden remain (3). The Global Burden of Disease (GBD) 2017 Study reported that cataract caused the second largest burden of disability-adjusted life years (DALYs) among eye diseases (8 million), next only to near vision loss (8). Meanwhile, the DALYs attributed to cataract increased by nearly 30% from 2007 to 2017 (8). To the best of our knowledge, existing studies assessing global cataract burden mostly focused on the cataract surgery rate (CSR) or cataract surgical coverage (CSC), (9, 10) few studies have specifically assessed the global change in cataract-related visual impairment burden and associated risk factors thus far (11, 12).

By 2030, the number of people worldwide aged 60 years and over is estimated to increase by 1.4 billion. Given most people over the age of 60 years will develop a cataract, the number of people with this condition will also increase substantially (13). A successful response to managing this impending challenge requires timely, reliable, quantitative information to design effective interventions aimed at counteracting the disease burden of cataract. Based on the currently available data on the global scale from the GBD, the present study aimed to provide updated information on the global burden of cataract to better inform key stakeholders contributing to the implementation of future health policies.

## Methods

### Data sources

The disease burden due to cataract (prevalence of visual impairment and DALYs) was obtained from the Global Disease Burden 2019 in the Global Health Data Exchange (GHDx) (http://ghdx.healthdata.org/gbd- results-tool), where the global burden of 369 diseases and injuries of 204 countries during 1990 and 2019 and territories were presented and its details of methodology were described (14). The definition of DALYs is the sum of the years lost due to disability and the years lived with disability by the following formula: DALY number = (Number of deaths × Standard life expectancy at age of death in years) + (Number of prevalent cases × Disability weight) (14, 15). The prevalence of visual impairment due to cataract and DALYs of cataract was calculated for populations aged 20 years and above, and the DALYs rate was adjusted for population size, with age-standardized DALYs rate further adjusted for age structure.

Further analyses were conducted using the following data: (1) the global, GBD regions, and GBD super regions numbers, rates, and age-standardized rates of DALYs and prevalence of visual impairment due to cataract from 1990 to 2019; and (2) country-level age-standardized DALYs rate of 2019.

### Country-level indicators

In order to assess the correlation between the disease burden of cataract and potential associated factors, various country-level indexes were included for statistical analysis. Former studies gave the conclusion that some of the common ocular diseases, such as glaucoma and refractive error, are positively associated with the development of cataract (16–18). Meanwhile, individuals with diabetes mellitus are at a higher risk of developing cataract (19, 20). The prevalence of diseases mentioned earlier was extracted from the GBD 2019 study (14). Cataract surgical rate, defined as the number of cataract surgeries performed annually per million population, indicates the progress in cataract control and was also included for analysis (3, 21, 22).

In terms of included socioeconomic factors, the socio-demographic index (SDI) is a commonly used index to assess the social and economic development level of a country from GHDx, which classifies countries into five categories, namely high SDI, middle-high SDI, middle SDI, low-middle SDI, and low SDI. Another comparable indicator, the human development index (HDI), was extracted from the United Nations Development Programme's (UNDP) database (http://hdr.undp.org/en/data). HDI consists of four components across health, education, and income dimensions: life expectancy at birth, expected years of schooling, mean years of schooling, and gross national income per capita (GNI). The HDI ranged from 0 to 1, and countries were accordingly divided into four groups: very-high HDI (HDI 0.800 and above), high HDI (HDI 0.700–0.799), medium HDI (HDI 0.55–0.699), and low HDI (HDI 0.549 and below). Inequality-adjusted HDI was also adapted as it adjusted for the unequal distribution of the HDI within each country. Populations with at least some secondary education (aged 25 years and older) are an indicator for the evaluation of education level among one country in addition to the two components of HDI. The number of physicians, defined by the number of medical doctors per 10,000 population, reflects the healthcare level of a country.

### Statistical analysis

The results are composed of time trends of the prevalence of visual impairment due to cataract and DALYs numbers, DALYs rate, and age-standardized DALYs rate attributable to cataract. The global distribution of the prevalence of visual impairment due to cataract was downloaded from the GHDx (https://vizhub.healthdata.org/gbd-compare/). One-way analysis of variance (ANOVA) was performed to detect GBD regional as well as GBD super regional differences in age-standardized DALYs rate. A scatter plot with a regression curve was drawn to evaluate the correlation between age-standardized DALY rates and SDI. Univariate and stepwise linear regression analyses were used to investigate the potential correlation with common ocular and endocrine diseases and demographic, socioeconomic, and healthcare indicators. Variables significant at a level of *P* ≤ 0.2 in univariate regression were included in multiple stepwise regression analyses. Figures were generated using GraphPad Prism software (V.5.01, GraphPad Software; San Diego, California, USA), while statistical analyses were conducted using Stata MP 15.1 (Stata Corp LP, College Station, Texas, USA). A value of *P* < 0.05 was considered statistical significance unless otherwise specified.

## Results

### Time trends of disease burden attributable to cataract from 1990 to 2019

**Figure 1:**
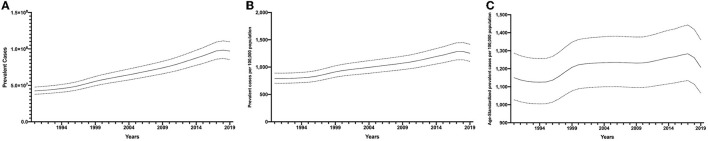
Global prevalence of visual impairment due to cataract from 1990 to 2019. Prevalence cases **(A)**, prevalence rate **(B)**, and age-standardized prevalence rate **(C)**.

**Figure 2:**
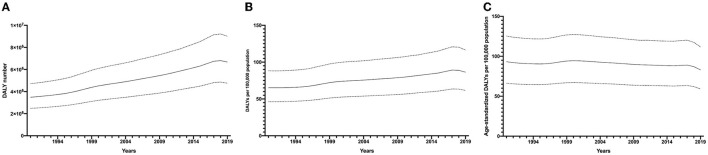
Global DALYs of cataract from 1990 to 2019. DALY numbers **(A)**, DALY rates **(B)**, and ae-standardized DALY rates **(C)**. DALYs, disability-adjusted life years.

The global trend of prevalence of visual impairment due to cataract and DALYs caused by cataract are presented in Figures 1, 2. The prevalence cases of cataract with visual impairment showed a steady increase of 129.2%, from 4,233, 6,679 (95% CI: 3,772, 8,563, 4,761, 5,115) in 1990 to 9,702, 2,038 (95% CI: 8,537, 0876, 1, 0969, 6,644) in 2019. The prevalence rate of visual impairment due to cataract also had an upward trend, rising by 58.45%, from 791.4 per 100,000 population (95% CI: 705.2 to 890.0 per 100,000 population) in 1990 to 1,253.9 per 100,000 population (95% CI: 1,103.3 to 1,417.7 per 100,000 population) in 2019. Age-standardized prevalence of visual impairment due to cataract fluctuated with its lowest in 1994 (1,125.55, 95% CI: 1,004.97, 1,256.61 per 100,000 population) and highest in 2017 (1,283.53, 1,134.46, and 1,442.93 per 100,000 population).

In general, trends of DALYs number and DALYs rate were similar to those of the prevalence of visual impairment due to cataract, as DALY numbers increased by 91.2% from 349, 2,604 (95% CI: 248, 1,846, 471.9629) in 1990 to 667, 6,281 (95% CI: 476, 1,210, 900, 6,193) in 2019. When it comes to the DALYs rate of cataract, it rose by 32.18% from 65.3 per 100,000 population (95% CI: 46.4 to 88.2 per 100,000 population) in 1990 to 86.3 per 100,000 population (95% CI: 61.5 to 116.4 per 100,000 population) in 2019. The age-standardized DALYs rate was uneven throughout the past 30 years with an overall declining trend, ending at 82.94 (95% CI: 59.06, 111.75 per 100,000 population) in 2019.

### Global distribution of disease burden attributable to cataract in 1990 and 2019 and its annual change

**Figure 3:**
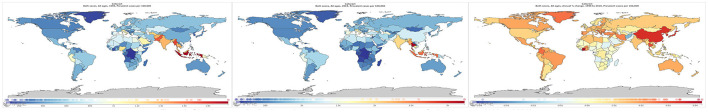
Global age-standardized prevalence of visual impairment due to cataract for both sexes in 1990 and 2019.

Figure 3 shows the global distribution of the prevalence of visual impairment due to cataract in the years 1990 and 2019. The countries in south Asia and south-east Asia tend to have a higher prevalence of visual impairment due to cataract in 1990 and 2019. Indonesia had the most severe disease burden in the year 1990 (1,972.50, 95% CI: 1,799.04, 2,158.74 per 100,000 population), while Thailand surpassed Indonesia in 2019 (3,231.99, 95% CI: 2,960.79, 3,497.79 per 100,000 population). The annual change for the prevalence of visual impairment due to cataract was positive in most countries, except for some countries located in the Middle East and Africa. As for the comparison of age-standardized prevalence of visual impairment due to cataract and DALYs rate between 1990 and 2019 among GBD super regions (Table 1), the global prevalence of visual impairment due to cataract in 2019 raised by 4.98% (95%CI: 4.86%, 5.11%) compared to that in 1990. The disease burden of most geographical regions tended to decline, with the exception of the east Asia region, where the DALYs rate increased by 14.13% (95% CI: 13.89%, 14.36%) to 967.90 (95% CI: 840.60, 1,104.44) per 100,000 population in the year 2019. The age-standardized DALY rates of 2019 among the selected GBD super regions, on the other hand, were all lower than those of 1990.

**Table 1:** Age-standardized prevalence of visual impairment due to cataract in 1990 and 2019 and its time trends from 1990 to 2019.

**Location**	**Prevalence (95% UI)**			**DALYS (95%UI)**		
	**1990**	**2019**	**Percentage change 1990–2019**	**1990**	**2019**	**Percentage change 1990–2019**
Global	1,150.56 (1,027.31, 1,287.40)	1,207.88 (1,065.04, 1,361.26)	4.98 (4.86, 5.11)	93.17 (66.14, 125.32)	82.94 (59.06, 111.75)	−10.98 (−11.63, −10.35)
Low SDI	2,016.86 (1,810.94, 2,241.28)	1,966.87 (1,753.94, 2,205.73)	−2.48 (−2.55, −2.41)	180.68 (128.63, 244.99)	154.48 (108.77, 206.59)	−14.50 (−15.02, −13.99)
Low-middle SDI	2,495.12 (2,234.99, 2,780.44)	2,182.06 (1,937.04, 2,455.86)	−12.55 (−12.68, −12.42)	216.76 (153.53, 292.32)	160.43 (114.24, 215.23)	−25.99 (−26.58, −25.40)
Middle SDI	1,634.59 (1,464.80, 1,816.28)	1,541.64 (1,373.65, 1,723.02)	−5.69 (−5.80, −5.57)	139.91 (99.06, 188.58)	107.61 (75.96, 144.28)	−23.09 (−23.79, −22.39)
High-middle SDI	775.10 (678.01, 876.07)	893.60 (774.42, 1,013.51)	15.29 (15.04, 15.54)	54.05 (38.25, 71.81)	53.72 (38.45, 72.57)	−0.61 (−0.86, −0.42)
High SDI	354.32 (305.76, 407.33)	357.01 (306.85, 409.46)	0.78 (0.69, 0.88)	21.86 (15.50, 29.71)	20.57 (14.49, 28.10)	−5.90 (−6.97, −4.95)
Central Europe	399.38 (335.02, 468.77)	391.55 (326.74, 462.98)	−1.96 (−2.10, −1.83)	21.26 (14.81, 28.96)	19.36 (13.34, 26.63)	−8.94 (−10.23, −7.76)
Australasia	356.57 (307.67, 406.66)	355.89 (301.23, 410.70)	−0.19 (−0.15, −0.02)	21.32 (14.83, 29.03)	20.30 (14.25, 27.82)	−4.78 (−5.78, −3.92)
Central Asia	1,219.74 (1,043.81, 1,397.11)	1,152.73 (977.42, 1,334.76)	−5.49 (−5.62, −5.37)	77.49 (54.76, 104.16)	66.22 (45.77, 89.99)	−14.54 (−15.35, −13.77)
Central Latin America	1,101.92 (966.45, 1,248.99)	942.18 (816.67, 1,077.83)	−14.50 (−14.71, −14.29)	90.64 (64.33, 121.65)	64.70 (46.00, 87.16)	−28.62 (−29.56, −27.69)
Tropical Latin America	1,143.56 (1,014.81, 1,280.30)	975.80 (857.20, 1,097.80)	−14.58 (−14.79, −14.38)	96.82 (68.48, 130.47)	70.86 (50.45, 94.07)	−26.81 (−27.71, −25.93)
Caribbean	690.17 (591.39, 796.14)	620.33 (525.97, 721.44)	−10.12 (−10.35, −9.90)	56.30 (39.63, 76.84)	42.90 (30.02, 58.37)	−23.80 (−24.94, −22.69)
Southern sub-Saharan Africa	1,082.82 (965.44, 1,207.09)	860.85 (763.12. 967.27)	−20.50 (−20.74, −20.26)	119.28 (84.16, 164.15)	80.48 (57.65, 110.15)	−32.53 (−33.38, −31.69)
Eastern Europe	531.04 (451.57, 618.71)	506.85 (428.34, 588.70)	−4.56 (−4.74, −4.38)	33.49 (23.53, 44.81)	28.94 (20.29, 39.35)	−13.59 (−14.79, −12.44)
Southern Latin America	564.67 (484.69, 649.62)	544.05 (462.34, 627.92)	−3.65 (−3.81, −3.50)	38.56 (26.81, 51.96)	32.50 (22.74, 44.10)	−15.72 (−16.90, −14.58)
Andean Latin America	1,764.55 (1,551.78, 1,988.38)	1,447.36 (1,266.22, 1,648.07)	−17.96 (−18.16, −17.80)	140.25 (99.00, 188.37)	96.22 (68.28, 129.43)	−31.39 (−32.17, −30.63)
Southeast Asia	3,110.31 (2,827.96, 3,422.57)	2,613.61 (2,391.13, 2,860.70)	−15.97 (−16.10, −15.84)	279.06 (198.51, 378.15)	195.67 (138.40, 262.38)	−29.88 (−30.42, −29.35)
Western Europe	448.19 (383.96, 519.03)	440.10 (376.20, 507.66)	−1.81 (−1.93, −1.68)	26.68 (18.64, 36.59)	25.31 (17.74, 34.81)	−5.13 (−6.04, −4.33)
High-income Asia Pacific	313.83 (271.38, 358.06)	310.34 (267.86, 356.64)	−1.11 (−1.23, −1.00)	21.09 (14.83, 28.65)	19.50 (13.59, 26.89)	−7.54 (−8.75, −6.45)
South Asia	3,098.49 (2,773.45, 3,456.87)	2,663.74 (2,359.84, 3,005.09)	−14.03 (−14.15, −13.91)	273.27 (194.60, 366.72)	198.39 (142.54, 264.15)	−27.40 (−27.93, −26.87)
High-income North America	289.16 (249.35, 330.20)	288.60 (249.38, 330.01)	−0.19 (−0.25, −0.15)	18.06 (12.84, 24.71)	17.42 (12.34, 23.78)	−3.54 (−4.50, −2.74)
East Asia	848.08 (744.35, 954.58)	967.90 (840.60, 1,104.44)	14.13 (13.89, 14.36)	63.90 (45.00, 86.66)	57.37 (40.44, 77.80)	−10.22 (−10.99, −9.49)
North Africa and Middle East	1,775.75 (1,566.58, 2,007.55)	1,534.09 (1,332.42, 1,756.61)	−13.61 (−13.77, −13.45)	143.15 (101.05, 195.59)	98.21 (69.44, 134.06)	−31.39 (−32.16, −30.63)
Oceania	2,467.72 (2,179.42, 2,784.63)	2,463.23 (2,174.16, 2,763.30)	−0.18 (−0.20, −0.17)	181.65 (128.82, 242.25)	163.72 (116.76, 222.47)	−9.87 (−10.31, −9.44)
Central sub-Saharan Africa	425.58 (365.03, 493.53)	413.80 (345.05, 487.15)	−2.77 (−2.93, −2.61)	32.86 (22.99, 45.35)	26.13 (18.05, 35.82)	−20.48 (−21.90, −19.11)
Eastern sub-Saharan Africa	1,651.44 (1,490.61, 1,822.16)	1,489.44 (1,337.15, 1,658.66)	−9.81 (−9.95, −9.67)	156.81 (112.06, 215.18)	129.15 (91.78, 176.19)	−17,64 (−18.24, −17.05)
Western sub-Saharan Africa	1,996.56 (1,798.53, 2,215.80)	2,155.19 (1,912.45, 2,412.08)	7.95 (7.83, 8.06)	165.62 (117.94, 224.66)	152.14 (107.71, 205.16)	−8.14 (−9.57, −7.73)

SDI, sociodemographic index; UI, uncertainty interval.

### Global cataract disease burden by SDI level

**Figure 4:**
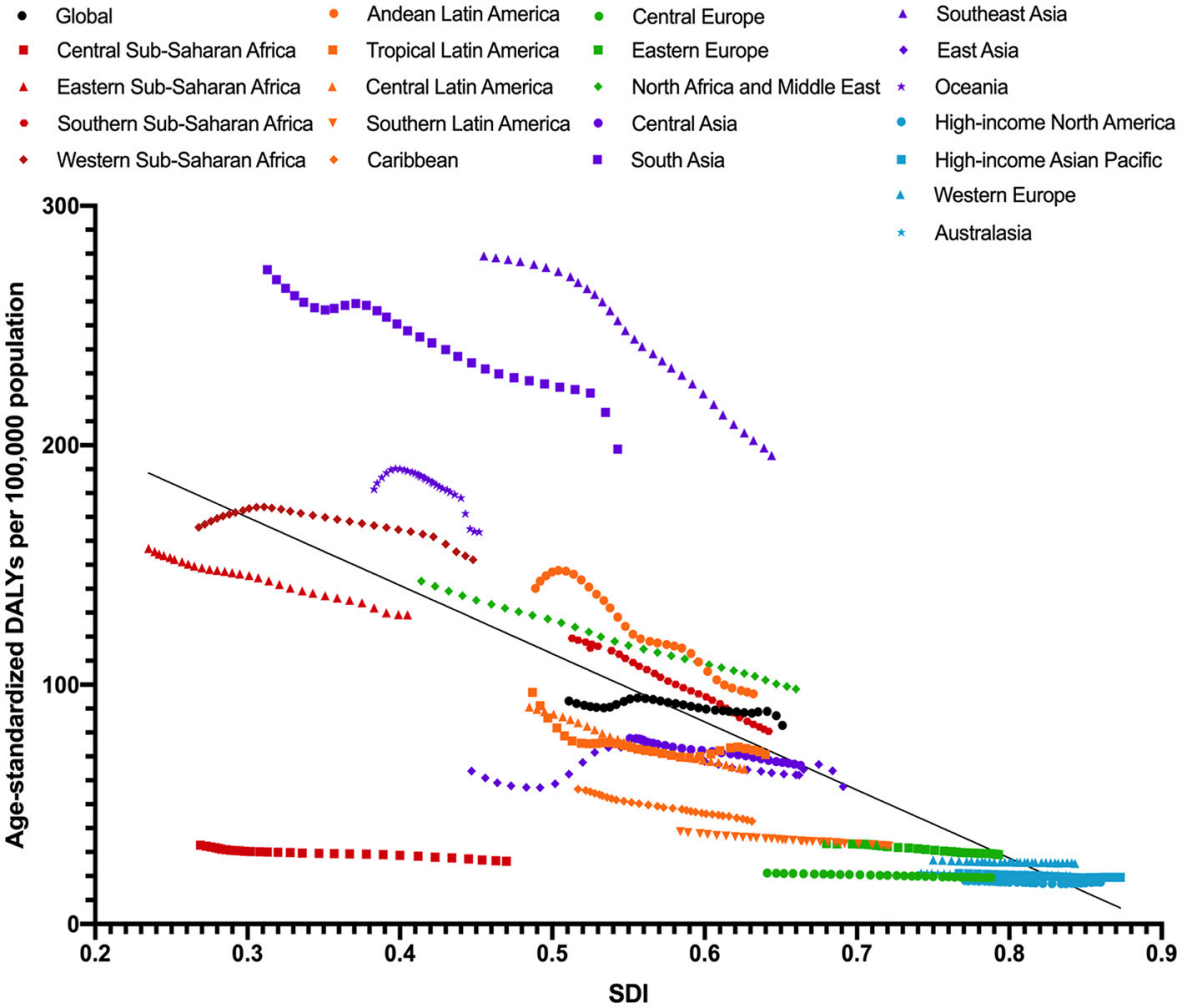
Age-standardized DALY rates of cataract across 21 GBD regions by socio-demographic index for both sexes combined, 1990 to 2019. DALY, disability adjusted life years; GBD, global burden of disease study.

The changes in age-standardized DALY rates among GBD super regions by SDI from 1990 to 2019 are given in Figure 4. Disease burden attributable to cataract in all regions tends to decrease throughout the past 30 years, with the region of higher SDI having less disease burden due to cataract. Individuals dwelling in southeast Asia and south Asia were at a higher risk of vision loss caused by cataract, whereas people in high-income North America, high-income Asian Pacific, Western Europe, and Australasia were less likely to develop vision loss due to cataract.

### Country-level analysis of disease burden attributable to cataract with various factors

As shown in Table 2, glaucoma and refractive error were positively associated with the disease burden of cataract, accounting for 16.95 and 14.30% of the global variation (*P* < 0.001), respectively. Diabetes mellitus, on the other hand, did not show any association with the disease burden of cataract.

**Table 2:** Potential associated factors for national disease burden due to cataract.

**Factors**	**Univariate linear regression**			**Multiple linear regression 1**		**Multiple linear regression 2**	
	β **(95%CI)**	***R** ^2^*	***P***	β **(95%CI)**	***P***	β **(95%CI)**	***P***
Glaucoma prevalence	0.279 (0.193, 0.364)	0.17	<0.001	-	-	-	-
Refractive error prevalence	0.038 (0.025, 0.051)	0.14	<0.001	0.036 (0.022, 0.050)	<0.001	0.032 (0.019, 0.046)	<0.001
Diabetes mellitus prevalence	0.002 (−0.0006, 0.004)	0.01	0.15	-	-	-	-
SDI	−159.55 (−197.16, −121.95)	0.26	<0.001	-	-	-	-
CSR	−0.006 (−0.010, −0.002)	0.06	0	-	-	-	-
Population with at least some secondary education	−1.101 (−1.380, −0.822)	0.28	<0.001	-	-	-	-
Number of Physicians	−1.678 (−2.156, −1.199)	0.13	<0.001	−0.959 (−1.685, −0.233)	0.01	−0.982 (−1.587, −0.196)	0.01
HDI	−86.161 (−123.207, −49.115)	0.11	<0.001	−134.93 (−209.84, −60.02)	0	-	-
IA-HDI	−80.531 (−106.725, −54.338)	0.18	<0.001	-	-	-	-
Life Expectancy at birth	−0.577 (−1.073, −0.081)	0.03	0.02	-	-	-	-
Expected years of schooling	−4.723 (−6.649, −2.797)	0.12	<0.001	-	-	−5.183 (−9.564, −0.802)	0.02
Mean years of schooling	−6.899 (−9.037, −4.761)	0.19	<0.001	-	-	−2.782 (−7.141, 1.577)	0.21
GNI per capita	−0.001 (−0.002, −0.0005)	0.11	<0.001	-	-	-	-

SDI, sociodemographic index; CSR, cataract surgery rate; HDI, human development index; IA-HDI, inequality-adjusted human development index; GNI per capita, gross national income per capita. Multiple linear regression 1: adjusted for glaucoma prevalence, refractive error prevalence, diabetes mellitus prevalence, CSR, population with at least some secondary education, number of physicians, and HDI. Multiple linear regression 2: adjusted for glaucoma prevalence, refractive error prevalence, diabetes mellitus prevalence, CSR, population with at least some secondary education, number of physicians, life expectancy at birth, expected years of schooling, mean years of schooling, and GNI per capita.

**Figure 5:**
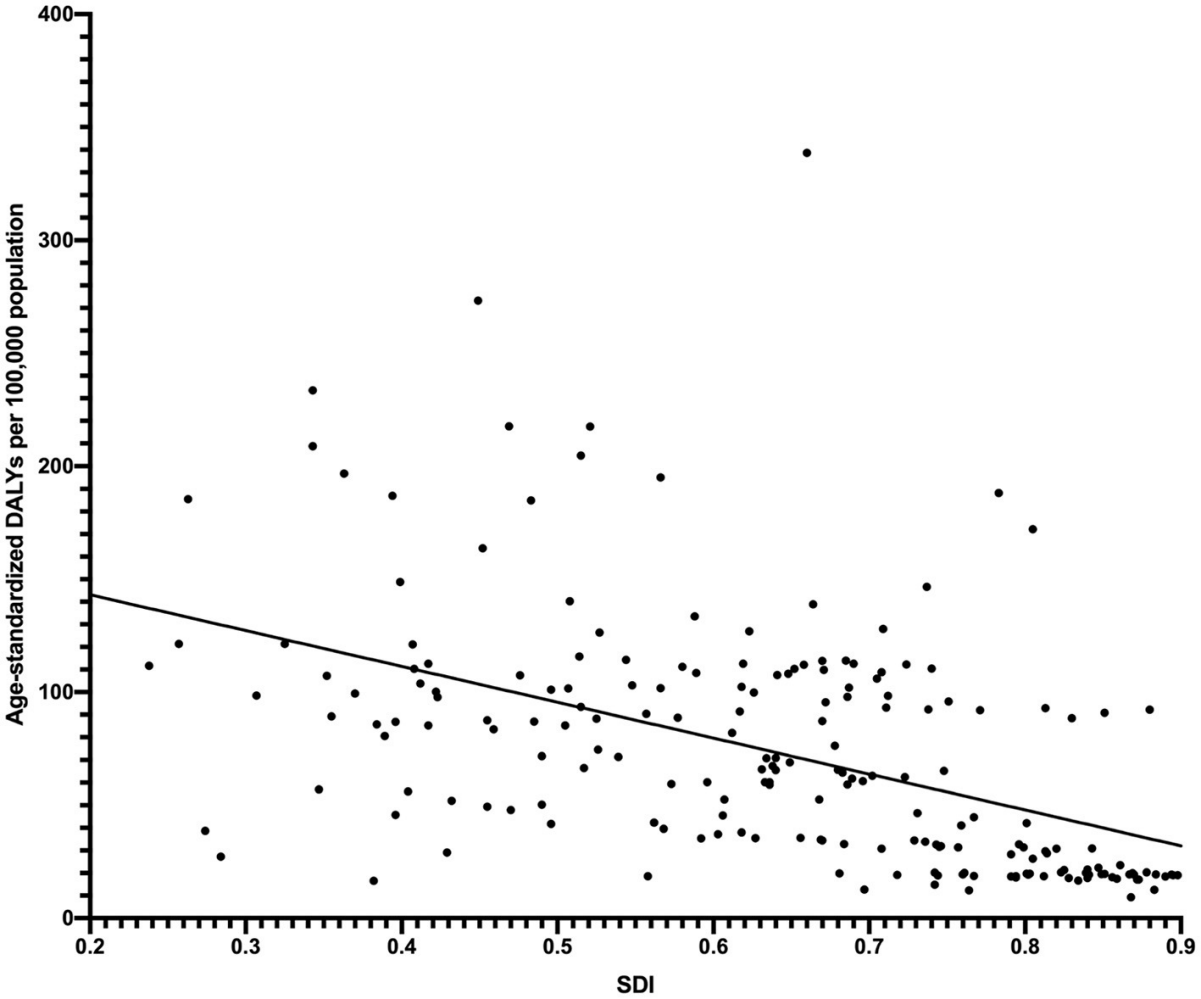
Age-standardized DALY rates of cataract across 204 countries and territories by socio-demographic index in 2019.

As for socioeconomic indicators, the scatter plot with regression curve indicated that the age-standardized DALY rate of cataract was negatively correlated to the SDI level (Figure 5). In the stepwise multiple linear regression model, higher refractive error prevalence (β = 0.036, 95% CI: 0.022, 0.050, *P* < 0.001), a smaller number of physicians (β = −0.959, 95% CI: −1.685, −0.233, *P* = 0.010), and lower level of HDI (β = −134.93, 95% CI: −209.84, −60.02, *P* = 0.001) were associated with a higher disease burden of cataract. Meanwhile, higher refractive error prevalence (β = 0.032, 95% CI: 0.019, 0.046, *P* < 0.001) and a smaller number of physicians (β = −0.982, 95% CI: −1.587, −0.196, *P* = 0.012) were also correlated with more severe disease burden in multiple regression model 2, together with shorter expected years of schooling (β = −5.183, 95% CI: −9.564, −0.802, *P* = 0.021).

## Discussion

Our results show that the global prevalence of visual impairment due to cataract and DALYs of cataract showed an increasing trend from 1990 to 2019, with a higher burden found in low socio-economic areas. A severe burden of visual impairment due to cataract is associated with lower SDI, lower HDI, smaller number of physicians, and higher refractive error prevalence. Our findings are consistent with previous GBD studies (11, 12), and further, emphasize the importance of implementing strategies to increase coverage and reduce gaps to eliminate the rising cataract burden globally.

The crude prevalence of visual impairment due to cataract and DALYs of cataract increased steadily from 1990 to 2017, but showed a decreasing trend afterward until 2019; this decreasing trend could more clearly be seen in age-standardized rates (Figures 1, 2). Meanwhile, despite the increase of age-standardized prevalence of visual impairment due to cataract by 4.98% during the past 30 years, the age-standardized DALY of cataract consistently decreased (decreased by 10.98%). Taken together, this suggests a global health improvement in cataract, especially after 2017. However, the overall global burden of cataract remains high, which can be partially explained by taking into account the aging and growth of the population.

Despite the overall improvement in global cataract care, the socioeconomic disparity in cataract burden is a major challenge for reducing global cataract blindness. Previous studies have reported higher cataract prevalence and lower cataract surgical rates in developing and undeveloped countries (23). The GBD studies have also reported consistently increasing socioeconomic-associated inequality in the global cataract burden in the last decades (12). In our study, the age-standardized prevalence of visual impairment due to cataract was highest in low-middle SDI regions and lowest in high-SDI regions in both 1990 and 2019. This could be attributed to better eye care services and few financial barriers in higher-income regions. In addition, cataract surgical outcomes in more developed countries are reported to be better (24). These collectively suggest that both quantity and quality should be emphasized to better address the socioeconomic disparity in the global cataract burden. The greatest declines in age-standardized DALYs rate of cataract between 1990 and 2019 were in Southern sub-Saharan Africa, Andean Latin America, and Southeast Asia, while the greatest increase was in East Asia and Western sub-Saharan Africa. With more than 20% of the total world population, the East Asia regions contribute to the largest increase in cataract burden and are in urgent need of future effective strategies to combat the upcoming increasing challenge of cataract burden. In addition, the age-standardized DALYs of cataract in 2019 was significantly reduced in all regions as compared to 1990, with the greatest decline seen in low-middle SDI regions. The percentage change in age-standardized DALY was largest in Southern sub-Saharan Africa, North Africa, the Middle East, and Andean Latin America (all over 30%). In comparison, the decreases in East Asia and Western sub-Saharan Africa were only modest (10.22 and 8.14%) and below the average global decreasing level.

Consistent with previous studies, our study showed that a lower number of physicians and less education were significant risk factors for higher cataract burden (25–27). It is noteworthy that higher refractive error prevalence was also found to be positively related to a higher national disease burden due to cataract, even after adjusting for all other covariates. One possible reason is that with the increasing prevalence of refractive error, the complex cataract cases due to myopia increase (28). Various studies have found that individuals with high myopia are more likely to develop cataract (28, 29). The Blue Mountains Eye Study found that even participants with low myopia (−1 to −3.5D) had a higher risk of posterior subcapsular cataract (30). A meta-analysis reported that myopia was associated with a higher risk of nuclear and posterior subcortical cataract (31). However, advances in surgical techniques for cataract, coupled with improvements in intraocular lens design and the increased availability of low-cost, high-quality intraocular lens, have led to significant improvements in patient accessibility and surgical outcomes. Over the last decades, the increase in the prevalence of visual impairment due to cataract and its associated disease burden warrants further investigation and monitoring of cataract on a national and global basis, as well as the identification of determinants for further increase. Implementation of currently known and effective treatments for cataract are expected to contribute significant productivity gains to the global economy at a fraction of the estimated costs to deliver them (32). Increasing the quantity and quality of cataract surgery services, and tailoring blindness-prevention programs to meet the needs of the local population and resources remain the most important solutions to the existing and impending cataract burden.

The present study provides stakeholders with estimates at the regional and global level over 20 years to help monitor the effectiveness of interventions regarding cataract through time. However, some limitations should be noted. The study was subject to the limitations that the GBD 2019 study had noted in its reports, including statistical assumptions and data sources (14, 33). Specifically for cataract, the use of aggregated data at a country level would be a source of bias, due to geographic variations in DALY estimates. In addition, potential associated factors for cataract burden including cost, ophthalmologist numbers, facilities, and surgical quality were not available (27, 34), and future studies investigating these important determinants for cataract burden can provide a more comprehensive assessment and support for strategic planning.

## Conclusion

In summary, cataract is still an important contributor to the global disease burden which is likely to continue to pose a great challenge in healthcare with population aging and growth. Successful global initiatives targeting improving cataract surgical rate and quality, especially in regions with lower socioeconomic status, is a prerequisite to combating this growing burden of cataract in the aging society.

## Data availability statement

Publicly available datasets were analyzed in this study. This data can be found here: https://vizhub.healthdata.org/gbd-results/.

## Author contributions

The study was designed by GJ and DZ. Conducting the study, data collection, and analysis and interpretation were completed by XH, MZ, ZL, YS, and CY. All authors contributed to the article and approved the submitted version.
